# Adherence to hepatitis A and hepatitis B multi-dose vaccination schedules among adults in the United Kingdom: a retrospective cohort study

**DOI:** 10.1186/s12889-019-6693-5

**Published:** 2019-04-15

**Authors:** Kelly D. Johnson, Xiaoyan Lu, Dongmu Zhang

**Affiliations:** 1 Center for Observational and Real World Evidence (CORE), Merck & Co., Inc., Kenilworth, NJ USA; 2 Center for Observational and Real World Evidence (CORE), Merck & Co., Inc., UG2AB-30, 351 N. Sumneytown Pike, North Wales, PA 19454 USA; 3 MSD Vaccines, Center for Observational and Real World Evidence (CORE), Lyon, France

**Keywords:** Hepatitis A, Hepatitis B, Hepatitis A/B, Multi-dose, Series completion, Vaccination

## Abstract

**Background:**

Timely and complete vaccination with multi-dose schedules is of public health importance, because an incomplete vaccination series may yield suboptimal disease protection. However, data on adherence of adults to multi-dose vaccines are limited. We sought to estimate adherence to multi-dose hepatitis vaccination schedules among adults in the United Kingdom (UK).

**Methods:**

This retrospective cohort study was conducted using anonymized electronic health record (EHR) data from the Clinical Practice Research Datalink (CPRD). Individuals aged 19 years and older at their first identified dose of hepatitis vaccine (2009–2016) were included if they had continuous EHR data for 12 months before the first identified hepatitis A dose or for 6 months before the first identified hepatitis B or combination hepatitis A/B dose. We estimated dose and series completion for each vaccine and adherence to recommended vaccination schedules, as well as adherence within additional prespecified time periods after the first vaccine dose, with sensitivity analyses restricted to adults who had available data for up to 24 months after the first dose. Median time to series completion was estimated using Kaplan-Meier methods.

**Results:**

Mean (SD) age at initiation was 42 (16) years for hepatitis A (*n* = 374,881), 40 (16) years for hepatitis B (*n* = 71,634), and 38 (15) years for hepatitis A/B (*n* = 10,335). Women comprised 52 to 55% of each vaccine cohort. Overall, 42,294 adults (11%) completed the two-dose hepatitis A vaccine series within the recommended 12 months; and 15,564 (22%) and 1076 (10%) completed the three-dose hepatitis B and hepatitis A/B series, respectively, within the recommended 6 months. These percentages rose to only 23, 35, and 33%, respectively, when the follow-up periods were extended to 36 months for hepatitis A and to 30 months for hepatitis B and A/B vaccines. Median times to series completion within recommended schedules were not reached in any cohort. Sensitivity analyses supported the primary findings for the full cohorts.

**Conclusions:**

Adherence and series completion rates for hepatitis A and B vaccines in the UK are low. Identifying, understanding, and addressing barriers to series completion for multi-dose vaccines for adults in real-world settings are needed.

**Electronic supplementary material:**

The online version of this article (10.1186/s12889-019-6693-5) contains supplementary material, which is available to authorized users.

## Background

Timely and complete vaccination with multi-dose schedules is of public health importance, because an incomplete vaccination series may yield suboptimal disease protection. Most studies investigating completion of multi-dose vaccine schedules have been conducted for pediatric and adolescent populations [1–3], for whom several different multi-dose vaccines are recommended. Instead little information is available regarding adherence to and completion of multi-dose vaccine series among adults.

Hepatitis A and hepatitis B are vaccine-preventable viral diseases of the liver [4–7] for which effective multi-dose vaccines have been available for many years. In the United Kingdom (UK), hepatitis A and/or hepatitis B vaccinations are recommended for adults at high risk of exposure to the specific virus or of complications from the disease [8, 9], among them travelers to high-risk countries, health care providers, intravenous drug users, sex workers, prisoners, patients with chronic liver disease, and any persons at risk of contact with blood or body fluids [10]. All recommended vaccinations are provided for free by the UK National Health Service (NHS). The usual recommended vaccination schedules are for two doses of the hepatitis A vaccine within 12 months, and for three doses of the hepatitis B or combination hepatitis A/B vaccine within 6 months.

Studies of adults in countries of low hepatitis prevalence, such as the UK, United States (US), Canada, and western Europe, indicate that hepatitis A and B vaccine series completion rates among adults are low [11–16]. For example, in a large 2015 survey in the US, only 16 and 32% of travelers to countries of high to intermediate endemicity had completed the two- or three-dose series for hepatitis A or hepatitis B, respectively [11]. The majority of studies of hepatitis vaccine series completion, however, are done in circumscribed populations such as the homeless [14], travelers [15], and adults with chronic liver disease [16].

There are few studies of series completion for multi-dose vaccinations in broad, general adult populations, and few studies have examined the rates of vaccine series completion within the recommended time frames [13], including none in the UK. We conducted a population-level analysis of vaccination completion rates using a large routinely collected dataset in the UK [17]. The primary objective of this study was to estimate dose and series completion for hepatitis A, hepatitis B, and combination hepatitis A/B multi-dose vaccines and adherence to the two- and three-dose vaccination schedules among adults in the UK receiving care under the umbrella of the NHS.

## Methods

### Data source

This retrospective cohort study used anonymized electronic health record (EHR) data from the Clinical Practice Research Datalink (CPRD), a large, well-managed database maintained by the UK Department of Health and Social Care [17]. Used frequently for pharmacoepidemiological research, the CPRD contains longitudinal EHR data that originates from over 600 subscribing general practices throughout the UK, representing about 5 million patients with active EHRs, or 7% of the UK population.

We used CPRD data from January 1, 2009, through December 31, 2016. The protocol for this study was approved by the CPRD Independent Scientific Advisory Committee (ISAC reference number 17_226R). No patient identifying information was accessible during the study.

### Study population

Adults who had a recorded dose of hepatitis A, hepatitis B, or hepatitis A/B vaccine at 19 years of age or older were eligible for the study. We required individuals receiving the hepatitis A vaccine to have at least 12 months of continuous baseline data before their first identified dose, while those receiving hepatitis B or hepatitis A/B vaccine were required to have at least 6 baseline months. The 12-month (hepatitis A) and 6-month (hepatitis B and A/B) baseline data requirements were selected to maximize the possibility that we identified the initiation of a vaccination series in the database rather than a booster.

Patients on hemodialysis who received an altered hepatitis B vaccination schedule or dosage were excluded from the study. In addition, individuals who received a second dose of the hepatitis A/B vaccine within 2 weeks of the first dose were excluded, as this schedule indicates use of accelerated administration requiring a total of four vaccines.

### Outcome measures

The primary study outcomes were (1) completion of the recommended number of doses of the relevant hepatitis vaccine (series completion) and (2) adherence to the recommended timing of the 2-dose hepatitis A and 3-dose hepatitis B and hepatitis A/B vaccines as per UK labeling for each vaccine product (Table 1) [18]. We calculated the proportions of adults who completed two and three doses and the proportions who adhered to the recommended schedule or within prespecified additional time periods after the first dose, as outlined in Table 1. In addition, we estimated the median time to completion of each dose and each vaccination series.

**Table 1:** Recommended adult administration schedules for hepatitis A, B, and A/B vaccine products in the United Kingdom

Vaccine	Recommended schedule			Additional study time points for completion assessments^a^
	Dose 1	Dose 2^a^	Dose 3^a^	
Hepatitis A	0	6–12 mo^b^	n/a	*Dose 2:* At 24 mo and 36 mo
Hepatitis B	0	1 mo	2–6 mo	*Dose 2:* At 13 mo and 25 mo
Hepatitis A/B	0	1 mo	6 mo	*Dose 3:* At 18 mo and 30 mo

^a^All times are relative to (after) the first dose

^b^Administration of the second dose of VAQTA® (Merck Sharp & Dohme, Hoddesdon, Hertfordshire, UK) is recommended at 6–18 months after the first dose [18], but for study purposes adherence was defined as 6–12 months

*mo* month; *n/a* not applicable (2-dose vaccine)

### Statistical analysis

The study population was stratified by first vaccine type (hepatitis A, B, or combination A/B), and each vaccine cohort was analyzed separately. Therefore, if an individual received both hepatitis A and hepatitis B vaccines during the study period, that individual would be included in both vaccine cohorts. Results for series completion and adherence were evaluated for the full cohorts and stratified by age at first dose within each cohort (19–49, 50–59, 60–64, 65–69, and ≥ 70 years). Baseline and outcome measures were summarized descriptively using means, medians, 95% confidence intervals (CIs), standard deviations, and frequency distributions for categorical variables.

We used Kaplan-Meier (KM) methods to estimate the time to completion of the second and third dose (as appropriate) at prespecified time points after series initiation. Only individuals with complete information for all the variables in the model were included in the study; hence there was no imputation for missing data. The use of the KM method allowed for inclusion of full vaccine cohorts by right-censoring of records for those without evidence of series completion. These individuals were censored at the end of the data. Survival function by age group was compared with the reference group of oldest adults (age ≥ 70 years) by calculation of hazard ratios (HR) with 95% CI.

We conducted sensitivity analyses to assess the impact of requiring a longer continuous baseline (pre-vaccine) period of 24 months’ data for the hepatitis A cohort and 18 months’ data for hepatitis B and hepatitis A/B cohorts. In addition, we analyzed series completion for individuals in each vaccine cohort with continuous follow-up data after the first vaccine dose of 6, 12, 18, and 24 months.

## Results

### Vaccine cohorts

The numbers of adults initiating a hepatitis vaccine series and eligible for the study are depicted in Additional file 1, Figure S1, and were greatest for hepatitis A (*n* = 374,881), followed by hepatitis B (*n* = 71,634) and hepatitis A/B (*n* = 10,335). Most individuals in each vaccine cohort were 19 to 49 years old, with mean ages of 42, 40, and 38 years in hepatitis A, B, and A/B cohorts, respectively (Table 2). Women comprised just over half of each cohort. The regional proportions of individuals in each cohort were greatest in London, with similar proportions throughout the UK in hepatitis A and hepatitis B cohorts, while the hepatitis A/B cohort had lower proportions in Scotland and Wales and greater proportions in some regions of England (see Table 2). From 55 to 58% of individuals in each cohort had available data of > 36 months in the CPRD after their first vaccine dose.

**Table 2:** Baseline characteristics of adults initiating a multi-dose hepatitis vaccination series from 2009 to 2016 in the UK

	Hepatitis A (*N* = 374,881)	Hepatitis B (*N* = 71,634)	Hepatitis A/B (*N* = 10,335)
Sex, n (%)			
Male	173,766 (46.4)	32,111 (44.8)	5001 (48.4)
Female	201,111 (53.6)	39,521 (55.2)	5333 (51.6)
Unknown	4 (0)	2 (0)	1 (0)
Mean (SD) age at initiation, years	42.0 (15.7)	39.8 (15.5)	37.9 (15.0)
Age distribution, n (%)			
19 to 49 years	251,070 (67.0)	51,739 (72.2)	7814 (75.6)
50 to 59 years	61,859 (16.5)	10,760 (15.0)	1405 (13.6)
60 to 64 years	25,895 (6.9)	3836 (5.4)	491 (4.8)
65 to 69 years	18,716 (5.0)	2552 (3.6)	375 (3.6)
≥70 years	17,341 (4.6)	2747 (3.8)	250 (2.4)
Government office region, n (%)			
North East	4852 (1.3)	1035 (1.4)	129 (1.2)
North West	35,114 (9.4)	7735 (10.8)	496 (4.8)
Yorkshire & the Humber	6068 (1.6)	1843 (2.6)	674 (6.5)
East Midlands	6101 (1.6)	1050 (1.5)	131 (1.3)
West Midlands	34,346 (9.2)	6037 (8.4)	996 (9.6)
East of England	28,054 (7.5)	5469 (7.6)	1602 (15.5)
South West	28,706 (7.7)	6006 (8.4)	1356 (13.1)
South Central	43,778 (11.7)	8230 (11.5)	691 (6.7)
London	58,582 (15.6)	14,124 (19.7)	1964 (19.0)
South East Coast	42,440 (11.3)	6105 (8.5)	1180 (11.4)
Northern Ireland	10,446 (2.8)	1450 (2.0)	226 (2.2)
Scotland	33,459 (8.9)	4185 (5.8)	251 (2.4)
Wales	42,935 (11.5)	8365 (11.7)	639 (6.2)
Months of continuous data after initiation			
Median (IQR)	43 (21–68)	40 (19–64)	39 (19–64)
Distribution, n (%)			
0–5 months	18,655 (5.0)	4048 (5.7)	593 (5.7)
6–11 months	30,272 (8.1)	6243 (8.7)	889 (8.6)
12–23 months	57,213 (15.3)	11,442 (16.0)	1682 (16.3)
24–35 months	52,238 (13.9)	10,238 (14.3)	1508 (14.6)
≥36 months	216,503 (57.8)	39,663 (55.4)	5663 (54.8)

*IQR* interquartile range

### Hepatitis A vaccine adherence and series completion

**Fig. 1:**
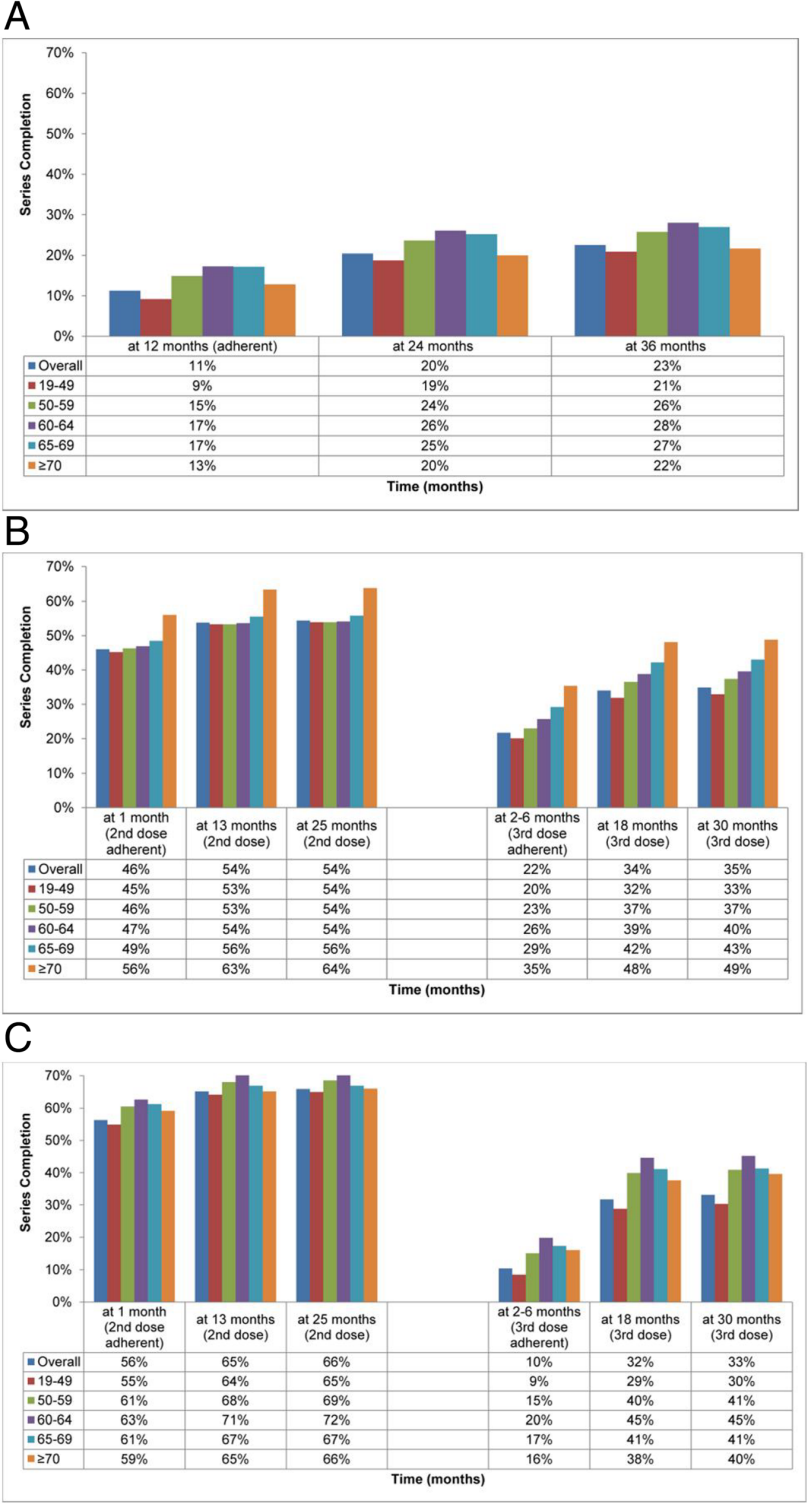
Vaccine adherence and series completion rates overall and by age group for (**a**) hepatitis A vaccine, (**b**) hepatitis B vaccine, and (**c**) hepatitis A/B vaccine

A total of 42,294 adults (11%) completed the second dose of hepatitis A vaccine within the recommended 12 months. These numbers increased to 76,649 (20%) at 24 months and to 84,337 (23%) at 36 months of follow-up after the first vaccine dose. Figure 1a depicts the series completion rates overall and stratified by age at 12, 24, and 36 months. The percentages of individuals completing the hepatitis A vaccine series within each prespecified time period were greatest in the three age groups from 50 to 69 years.

**Fig. 2:**
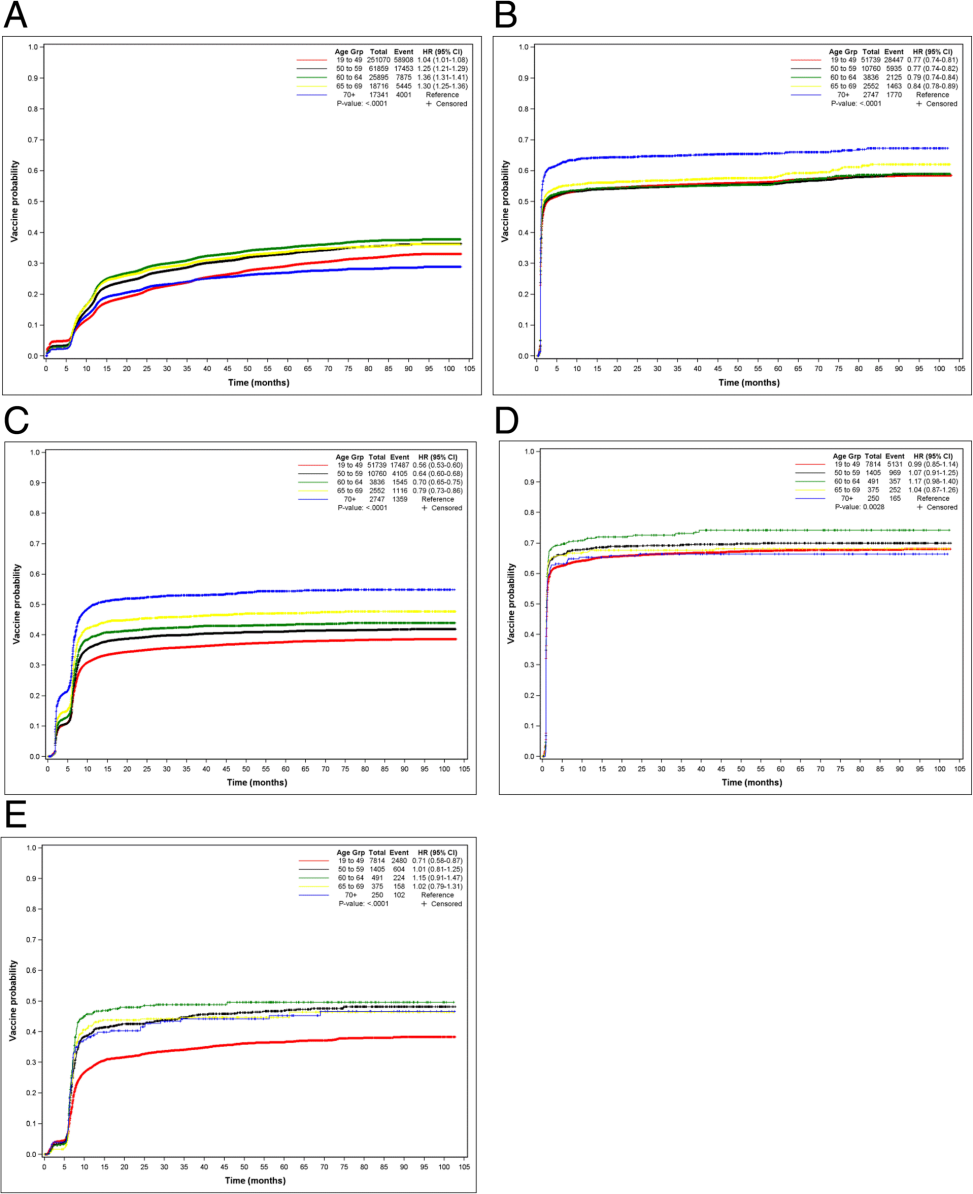
Kaplan-Meier curves depicting time to series completion after initiation of each vaccine, by age group for the (**a**) second hepatitis A dose, (**b**) second hepatitis B dose, (**c**) third hepatitis B dose; (**d**) second hepatitis A/B dose, and (**e**) third hepatitis A/B dose. *Age Grp* age group; *HR (95% CI)* hazard ratio (95% confidence interval)

The KM curves for the time to completion of the second dose of hepatitis A vaccine are shown in Fig. 2a by age group. The KM estimate of median time (in months) to series completion was not reached (NR; interquartile range [IQR] 33.1–NR; 75% of persons censored).

### Hepatitis B vaccine adherence and series completion

Overall, 32,951 adults (46%) were adherent with the schedule for the second dose of hepatitis B vaccine (received by 1 month after the first dose), and 15,564 (22%) were adherent with the third dose (from 2 to 6 months after the first dose). Older adults ≥70 years had the greatest adherence to this schedule, with 56 and 35% receiving the second and third doses by 1 month and 2–6 months, respectively (Fig. 1b).

By 13 months after the first dose, 38,500 individuals (54%) overall had received the second dose, numbers that by 25 months increased only slightly to 38,953 (54%). For the third dose, 24,344 (34%) and 25,016 (35%) completed the series by 18 and 30 months, respectively. The oldest age stratum maintained the greatest adherence to second and third doses over these longer follow-up periods (Fig. 1b).

The KM estimate of median time to completion of the second dose of hepatitis B vaccine was 2.3 months (95% CI 2.1–2.5; 45% of persons censored). The median KM time (in months) to series completion (third dose) was not reached (NR; IQR 6.97–NR; 64% of persons censored). There was little change in the KM curves by age for the second dose after about 5 months (Fig. 2b) and for the third dose after about 12 months (Fig. 2c).

### Hepatitis A/B vaccine adherence and series completion

Adherence to the hepatitis A/B recommended schedule was 56% for the second dose (*n* = 5822) by 1 month, and 10% for series completion (receipt of the third dose; *n* = 1076) by 6 months. As for the hepatitis A vaccine, adherence was greatest in the three middle age groups (50 to 69 years; Fig. 1c).

Completion of the second dose was achieved by 6739 adults (65%) by 13 months and by 6807 (66%) by 25 months. Completion of the third dose was achieved by 3276 adults (32%) by 18 months, increasing by 1% to 3417 (33%) by 30 months, again with the greatest percentages of series completers being in the 50 to 69-year-old age groups.

The median time to completion of the second dose, estimated by the KM method, was 1.2 months (95% CI 1.2–1.2; 33% of persons censored). The median KM time to series completion (third dose) was not reached (NR; IQR 7.87–NR; 65% of persons censored). The KM curves for completion of second and third doses by age group are depicted in Fig. 2d and e.

### Sensitivity analyses

Limiting the analyses to adults with 24 months of continuous baseline enrollment before initiation resulted in a decrease of < 1% in adherence to the second dose of hepatitis A vaccine, whereas adherence to the second and third doses of hepatitis B and hepatitis A/B increased slightly by < 1 to 2% with the requirement of 18 months’ continuous baseline enrollment (Table 3).

**Table 3:** Adherence to recommended vaccine schedules for all adults included in the study and for those included in sensitivity analyses with prespecified lengths of baseline (pre-vaccine) data and follow-up data after the first vaccine of the series

		Baseline	Continuous data available in follow-up period			
Adherence	All adults	18/24-mo^a^	6 months	12 months	18 months	24 months
Hepatitis A, n	374,881	279,451	353,880	322,533	293,056	264,522
To 2nd dose, n (%)	42,294 (11.3)	30,020 (10.7)	42,266 (11.9)	41,056 (12.7)	38,134 (13.0)	34,851 (13.2)
Hepatitis B, n	71,634	52,734	67,076	60,685	54,872	49,142
To 2nd dose, n (%)	32,951 (46.0)	24,398 (46.3)	31,217 (46.5)	28,160 (46.4)	25,450 (46.4)	22,719 (46.2)
To 3rd dose, n (%)	15,564 (21.7)	11,588 (22.0)	15,321 (22.8)	13,888 (22.9)	12,574 (22.9)	11,251 (22.9)
Hepatitis A/B, n	10,335	7746	9673	8764	7869	7056
To 2nd dose, n (%)	5822 (56.3)	4525 (58.4)	5534 (57.2)	5024 (57.3)	4481 (56.9)	4004 (56.8)
To 3rd dose, n (%)	1076 (10.4)	840 (10.8)	1073 (11.1)	1002 (11.4)	895 (11.4)	808 (11.5)

^a^Sensitivity analysis requiring continuous baseline (pre-vaccine) data of 24 months for the hepatitis A vaccine series and of 18 months for the hepatitis B and hepatitis A/B vaccine series

Requiring longer periods of continuous enrollment after initiating the three vaccine series resulted in slightly higher completion and adherence, but results were within 2 percentage points of those for the full cohorts (Table 3).

## Discussion

The rates of adherence to and series completion of UK vaccination schedules for multi-dose hepatitis A and hepatitis B vaccines were low among adults in this retrospective observational study. The percentages of adults adhering to and completing the recommended multi-dose series were 11, 22, and 10% for hepatitis A, hepatitis B, and hepatitis A/B vaccines, respectively. These percentages rose to only 23, 35, and 33%, respectively, when the follow-up periods were extended to 36 months for hepatitis A and to 30 months for hepatitis B and A/B vaccines, well beyond the recommended windows for vaccine series completion. Sensitivity analyses requiring longer periods of continuous data before and after vaccine series initiation supported the primary findings, with adherence rates within 2 percentage points. Overall, these analyses suggest substantial waste of health care resources, with vaccines delivered but likely suboptimal protection obtained because of incomplete vaccination series.

We found that individuals in the older age groups (≥50 years old) tended to be more adherent than younger adults (19–49 years old). This may be because older adults received the hepatitis vaccine together with an influenza vaccine, recommended annually in the UK for individuals 65 years and older, or together with the shingles vaccine, recommended for adults at age 70 [10]. Both influenza and shingles vaccine are administered at no cost under UK NHS care [19]. The hepatitis A vaccine is recommended most for travelers, while hepatitis B is recommended for both travelers and health-care providers, which could explain the considerably better adherence rates for the second dose of the hepatitis B vaccine (46%) and combination hepatitis A/B vaccine (56%), as compared with the second dose of hepatitis A vaccine (11%).

Few studies have examined multi-dose vaccine series completion in general adult populations. The recent study of Trantham et al. [13], designed similarly to the present study but using administrative claims data, reported somewhat higher, but still poor, rates of adherence in the US than in the UK, namely, rates of adherence to recommended schedules of 27, 30, and 18% for hepatitis A, hepatitis B, and hepatitis A/B, respectively. In addition, similar to the present study, they found that older adults had better adherence rates. The earlier study of Nelson et al. [20] in the US also found better adherence among older adult age groups and, for those adherent in all age groups, relatively long intervals between vaccine doses as in the present study and others [13].

Prior studies have reported suboptimal rates of series completion for hepatitis vaccine also in defined subpopulations, including patients with chronic liver disease [16], former prisoners [14], intravenous drug users [21], the homeless [22], and travelers [15, 23, 24]. Most of these studies did not examine series completion with regard to the recommended timelines.

Identification of the factors associated with adherence and dose/series completion was outside the scope of the present study; however, initiatives to improve patient and health care provider reminder systems, as well as opportunistic vaccination at other appointments, could be helpful to increase adherence and vaccination series completion. In a prior small study, Reynolds et al. [25] investigated factors associated with poor completion rates in a low-income minority population. They found that receipt of fewer doses of vaccine was associated with being male and having severe negative emotions, among other factors. The rate of three-dose series completions was only 31% despite the vaccinations being offered at no cost. Similarly, in our study, cost would not have been a potential barrier, because the NHS provides the vaccines for free. Offering financial incentives to encourage the return for second and third doses improved adherence in two studies of intravenous drug users [26, 27]; however, financial incentives were determined to not be a factor in series completion in a study of former prisoners [14]. Dedicated clinics have reported improved adherence when using multiple interventions, including SMS text reminders [28]. In addition, health-care provider recommendations to vaccinate are thought to be important, although studies find that providers, even at academic centers, often fail to adhere to hepatitis vaccination guidelines for patients with chronic liver disease [29, 30].

This study has several strengths. We used a large, high-quality database considered to have reliable capture of administered vaccinations [31] and that includes EHRs for a geographically diverse, representative UK population [32]. We studied almost half a million adults initiating hepatitis vaccination series, excluding patients receiving hepatitis B vaccination while on hemodialysis as they may not be representative of the general adult population. In addition, we used the KM method, used in few prior studies [13, 33, 34], to determine time to completion of second and third doses. The study design enabled us to identify the probable first vaccine dose by requiring baseline data periods of 6 to 12 months, extended to 18 to 24 months, depending on vaccine type, in sensitivity analyses. The long follow-up, including sensitivity analyses examining continuous data for up to 36 months after the first vaccine, enabled us to study series completion as well as to examine in detail whether these multi-dose vaccines were administered at the recommended intervals. Of course, patients could receive a booster vaccine even 5 years after the original course, although the results of our 36-month follow-up analyses suggest that overall findings would not change. However, we acknowledge that only 55–58% of patients had available data for ≥36 months after vaccine initiation.

A limitation of using the CPRD is that the EHR data are recorded for clinical purposes rather than specifically for research purposes. Moreover, as the CPRD data comprise EHRs from general practices, only the vaccinations received at those practices would be reliably recorded, while vaccinations administered in other settings such as hospitals, pharmacies, travel clinics, or occupational health services may have been missed. As for all observational studies, there is the potential for selection bias and possibility of unmeasured confounders. Another limitation is related to the comparison of completion for the selected two-dose and three-dose vaccines. The hepatitis A series has a longer window for completion than the combined A/B series, which could lead to higher completion within the recommended window. Finally, we did not examine, hence do not report, characteristics of the study population beyond sex, age, and UK region.

These limitations raise questions that would benefit from further study. In particular, a more complete capture to include all vaccinees from settings other than general practices and the reasons for these individuals initiating hepatitis vaccination series would be of great interest. We were unable to locate published population-level data for the UK regarding other settings, such as addiction or homeless services, and the reasons for hepatitis vaccinations (eg, for high-risk individuals, health care workers, travelers, etc.). As a corollary, it would be of interest to understand geographical vaccination patterns (eg, urban vs countryside) to identify areas of need. Finally, as noted above, better understanding of factors affecting adherence and of effective initiatives to improve adherence are needed to counter the potential waste of health care resources that occurs with suboptimal protection from incomplete vaccination series.

## Conclusions

Adherence and series completion rates are low for hepatitis A, hepatitis B, and hepatitis A/B multi-dose vaccines for adults in the UK. Series completion rates, even with extended observation windows, ranged from only 23 to 35%. The majority of adults initiating hepatitis vaccination series in the UK may not be receiving the full protective benefit of these multi-dose vaccines because of receipt outside the optimal time frame or failure to complete the series. Work is needed to identify, understand, and address barriers to series completion for adults receiving multi-dose vaccines in real-world settings.

## Additional file

Additional file 1: **Figure S1.** Flow chart depicting identification of individuals eligible for the study in the Clinical Practice Research Datalink (CPRD). (PDF 148 kb)

## Abbreviations

95% CI: 95% confidence interval
CPRD: Clinical Practice Research Datalink
EHR: Electronic health record
HR: Hazard ratio
IQR: Interquartile range
KM: Kaplan-Meier
NR: Not reached
UK: United Kingdom
US: United States
