# Supplementary material for: Adherence to hepatitis A and hepatitis B multi-dose vaccination schedules among adults in the United Kingdom: a retrospective cohort study
Source: BMC Public Health. 2019 Apr 15;19:404. doi: 10.1186/s12889-019-6693-5 (PMC6466685; doi:10.1186/s12889-019-6693-5)

Figure S1. Identification of eligible adults ≥19 years old initiating hepatitis A, hepatitis B, or hepatitis A/B vaccination series in the Clinical Practice Research Datalink (CPRD).

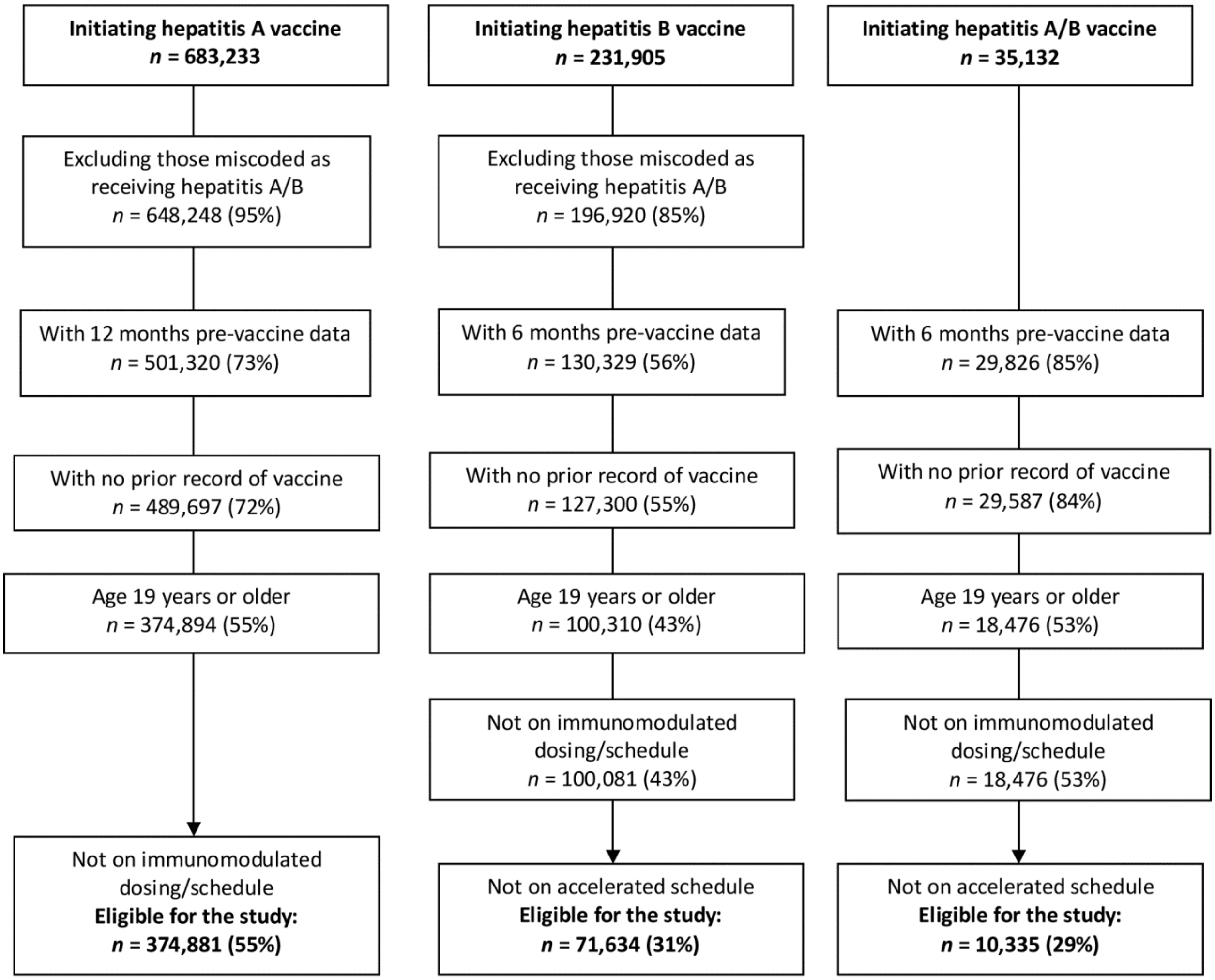

Supplement: Supplementary file 1 — Figure S1. Flow chart depicting identification of individuals eligible for the study in the Clinical Practice Research Datalink (CPRD). (PDF 148 kb) [file 12889_2019_6693_MOESM1_ESM.pdf]
